# Acoustic Performance of Tufted Carpets Coupled with Underlayment Produced from Tannery Wool Waste

**DOI:** 10.3390/ma18020315

**Published:** 2025-01-12

**Authors:** Jan Broda, Katarzyna Kobiela-Mendrek, Marcin Baczek, Monika Rom

**Affiliations:** Faculty of Materials, Civil and Environmental Engineering, University of Bielsko-Biala, Willowa 2, 43-309 Bielsko-Biala, Poland; kkobiela@ubb.edu.pl (K.K.-M.); mbaczek@ubb.edu.pl (M.B.); mrom@ubb.edu.pl (M.R.)

**Keywords:** wool, carpet, underlayment, sound absorption, transmission loss

## Abstract

Sheep wool is a precious, renewable raw material that is nowadays disregarded and wasted. To better use local sources of wool, it was used to manufacture tufted carpets. The coarse wool of mountain sheep was used to form a carpet pile layer, while the waste wool from the tannery industry was applied to form carpet underlayment. During investigations, the acoustic performance of the carpets was assessed. The carpets’ sound absorption coefficients and transmission loss were determined using the impedance tube. It was revealed that the adding of underlayment improves the carpet’s sound absorption only at medium sound wave frequencies. The underlayment significantly increases transmission loss in the whole frequency range. The acoustic performance of the carpets with the wool underlayment is similar to the acoustic characteristics of the carpets with an underlayment made from polyester. It was concluded that wool nonwovens can be used as an effective, eco-friendly, sound-absorbing carpet underlayment, which can improve wool utilisation and contribute to the reduction in environmental pollution caused by plastic residues.

## 1. Introduction

Industrial activities, road, rail, and air traffic, construction works, and many other human activities generate sounds, which are the source of environmental noise. Excessive noise has become a severe and pervasive pollutant that harmfully influences physical and mental health [1]. In addition to external noise, the inner noise in closed rooms has become a severe problem. Exposure to domestic noise negatively affects human well-being, impairs productivity, generates higher stress, and contributes to somatic complaints. Given the adverse effects of noise, the urgent need to reduce noise levels in homes, classrooms, open-plan offices, workplaces, and public facilities is highly significant and desirable today. 

For many years, various textiles used as window curtains, tapestries, upholstery, wall and ceiling panels, screens, rugs, and carpets have been used to reduce noise and improve the acoustic comfort of interiors [2,3,4,5]. Among these products, carpets are the most versatile, controlling indoor noise in various ways. First, carpets lower the level of noise by absorbing airborne sound. Second, they reduce the generation of floor impact sounds produced by footfalls, furniture movement, and objects dropped onto the floor. Third, carpets minimise the noise transmission through floors to adjoining rooms in multi-storied buildings.

Pile carpets, in particular, demonstrate a significant potential for noise reduction. These carpets are made of two layers that perform different functions and have different structures. The top layer, subjected to foot traffic, comprises overhanging yarn segments arranged in a compact system formed from loops or upright tufts. Instead of reflecting sound waves into the room, this layer allows their penetration into the piles. The penetrating waves cause the vibration of air molecules between both the individual fibres and pile tufts. The oscillating air molecules rub against irregular interstices and lose their energy due to friction. Eventually, the sound wave’s energy is transformed into thermal and viscous heat, which is finally dissipated. The energy dissipation in the pile layer is most effective at high sound frequencies [6]. 

The sound absorption in the pile layer depends on the pile type (loop or cut) and its height and density. Due to the open fuzzy structure, carpets with cut piles have higher sound absorption capacity than carpets with loop piles. Increasing pile height and density increases the interphase contact surface between piles and vibrating air molecules. Then, the energy dissipated by friction is higher and the resulting sound absorption increases [7,8,9]. The influence of other parameters characterising the pile layer, such as fibre type, yarn parameters, or knot type in knotted-pile carpets, is less distinguishable and difficult to estimate [10]. 

The second layer, the carpet base, provides carpets with appropriate dimensions and dimensional stability. The base consists of a woven fabric for woven and knotted-pile carpets. For carpets manufactured by tufting, the base is a multi-layered composite built from the primary backing, a layer of adhesive compound, and secondary backing fabric. The primary backing, formed from fabric with a mesh structure, is the carpet’s foundation, which locks the tufted yarns inserted by a needle. The secondary backing covers the loops of the tufted yarns, protects the tufts from being pulled out, and enhances the carpet’s final dimensional stability, strength, and stretch resistance. The base’s structure and the finishing type affect the absorption and transmission of sounds and significantly contribute to the carpet’s overall acoustic properties. The acoustic performance of the base, like that of all other weave fabrics, depends on the weave structure and other structural parameters, including cover factor and yarn density [11]. The base, which possesses distinct geometry and is formed from fibres other than the pile layer, has different resonant frequencies in which it absorbs sound. Consequently, the maximal sound absorption falls in other ranges, potentially improving the sound absorption coefficients at low and medium sound frequencies. The sound absorption at lower frequencies is enhanced for a layered base formed from two woven fabrics separated by an air gap [12]. 

In addition to the two essential layers, top and base, certain carpets are coupled with additional underlayment formed from nonwovens, felt or foam. The underlayment provides a barrier between the carpet and the floor, cushions footfalls, and extends carpet life. Simultaneously, it secures carpets with better thermal insulation and significantly reduces airborne and structure-borne noise. Adding underlay increases the sound absorption, principally in the mid-frequency range [13]. Soft underlay effectively lessens the force of the impact of footsteps and objects dropped onto the floor. The underlay layer reduces impact noise and its transmission through the floor, significantly contributing to the room’s soundproofing.

Pile carpets are manufactured from various natural and synthetic fibres. Although synthetic products dominate today’s market, the most valued and luxurious carpets are made from sheep wool [14]. These carpets possess high aesthetic values and are distinguished by high durability, flame resistance, and other beneficial functional properties [15,16,17,18].

A coarser type of wool, the so-called carpet wool, is applied to produce carpets [19,20]. The thickness of this wool exceeds 35 µm, making it, contrary to Merino wool and other fine and soft wool types, useless for producing apparel textiles. The appropriate wool, which meets manufacturing requirements and ensures superior carpet appearance and performance, is sourced worldwide from different sheep breeds. Often, suitable wool is obtained from local breeds, which deliver wool that is nowadays undervalued by the market and frequently treated as a waste of sheep husbandry [21,22]. 

In ordinary practice, wool, which is a sustainable, renewable, and biodegradable raw material, is used to produce rug yarns designed to construct only the pile layer. The remaining carpet layers, base, and underlayment are made from other natural or synthetic materials. Due to their excellent mechanical properties, chemical and biological resistance, and low price, fabrics made from synthetic fibres, mainly polyester or polypropylene, are often applied as the base. As the underlayment, polyurethane or polyethylene foams or polyester nonwovens are used primarily. Applying synthetic materials generates non-biodegradable waste and contributes to the pollution of the environment with plastic residues. For the sake of environmental concern, reducing the amount of plastic and replacing it with biodegradable materials is highly desirable [23].

To reduce the application of plastic and eliminate largely unexploited wool waste, investigations into the use of wool to produce carpets were performed. In previous investigations, coarse wool from Polish mountain sheep was applied to produce a pile layer of tufted carpets. The rug yarns were manufactured, and carpets with cut and loop piles were obtained. The investigations revealed that the acoustic properties are determined by the pile type and height and the density of the pile layer [24,25]. As a continuation of these investigations, studies of the acoustic performance of carpets coupled with additional underlayment were carried out. The underlayment was made from tannery waste wool. This wool is obtained by dehairing the sheep hide as a by-product of the tanning processes [26]. The wool is too short to be used in textile processes and, in many countries, is treated as troublesome and useless waste. A few papers report attempts to use this waste to produce thermal insulating composites or fertilisers [27,28].

Needle-punched nonwovens from waste tannery wool were obtained and applied as carpet underlayment. The effectiveness of the wool underlayment in sound absorption and transmission loss of the carpet was compared with the performance of products coupled with polyester nonwoven fabrics.

## 2. Materials and Methods

### 2.1. Materials

The investigations were carried out on the carpets, consisting of three layers: pile layer, base, and underlayment. 

The pile layer and the carpet base were obtained similarly as in studies reported in previous papers [24,25]. The piles were made from the coarse wool of Polish mountain sheep raised in the Silesian Beskid Mountains in southern Poland. The wool was collected during shearing and separated from heavily contaminated wool and wool of worse quality, designed to be used as mulch or fertilisers. The wool is a mixture of thinner, shorter, and more delicate fibres with diameters varying between 35 and 40 µm, thicker and longer guarded hairs containing a significant amount, ca. 10%, of medullated fibres, and kemp [29,30]. 

The wool was used to manufacture semi-worsted ring-spun three-plied yarn with a linear density of 1240 tex. The yarn was manufactured in the Selbu Spinneri (Klæbu, Norway), using a small-scale technological line of carding, drafting, and spinning machines set by Belfast Mini Mills (Prince Edward Island, Canada) and Ramella (Biella, Italy). 

The yarn was inserted into the carpet base using a manual pneumatic tufting gun in industrial conditions in the Sztuka Beskidzka (Czechowice-Dziedzice, Poland). The yarns were punched with a tubular needle into a pre-stretched primary backing (PB) mounted on a vertical frame. Then, a secondary backing (SB) was bonded with 520 g/m^2^ latex adhesive, commonly used for finishing tufted carpets. A plain polyester weave cloth with a square set of 70 dm^−1^ and yarn linear density of 180 tex was applied as the primary backing. As the secondary backing, a leno woven fabric with a mesh structure consisting of polypropylene warp with a linear density of 33 tex and set equal to 64 dm^−1^ and polyacrylonitrile weft with a linear density of 125 tex and set equal to 40 dm^−1^ was used. 

Two sets of carpets with cut and loop piles with a height of 12 mm were manufactured. The tuft density for carpets with cut piles was about 42·10 dm^−2^. For loop piles, the density was lower, approximately 40·10 dm^−2^.

Three nonwovens of different masses per unit area made from waste wool were applied as carpet underlayment. The wool was a waste product from the tanning industry and a mixture of fibres of various lengths and thicknesses from different Norwegian sheep breeds. The nonwovens were manufactured using a needle-punching technique by interlocking the carded web with the one-side needling machine with a punching density of 28 cm^−2^. The nonwovens were produced in industrial conditions, using that technological line (Jiangsu XST Machinery Technology, Wuxi, China).

Additionally, the commercial needle-punched nonwoven made from polyester, commonly used as carpet underlayment, was applied as a control. 

**Figure 1:**
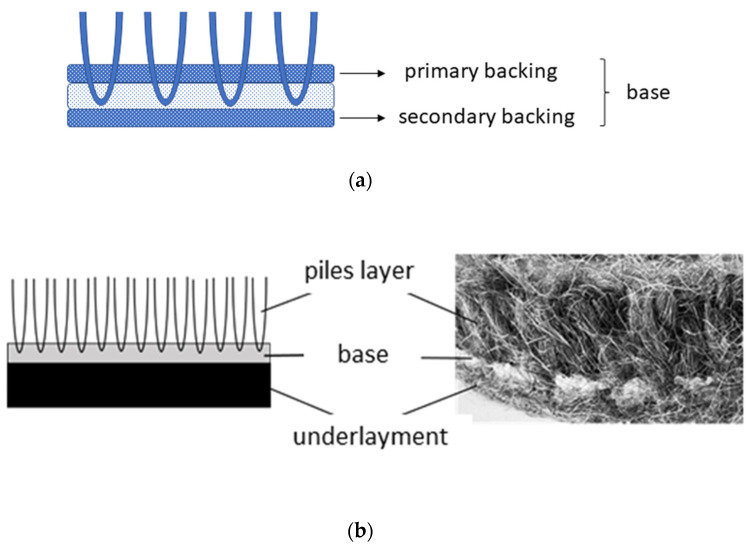
The carpet construction: (**a**) the base layer; (**b**) the composite multi-layered structure.

Finally, the multi-layered sandwich structures were produced by bonding the underlayment with latex adhesive (Figure 1). 

### 2.2. Methods

Surface density and thickness were measured to characterise nonwovens used as carpet underlayment. The measurements were carried out according to Polish standards PN-EN ISO 9863-1:2007 and PN-EN ISO 9864:2007. The thickness was measured under a 2 kPa load using a Tilmet 73 thickness gauge (Lodz, Poland). Each parameter was determined as the mean of 10 measurements. 

The morphology of the nonwovens was studied using Scanning Electron Microscopy. Before the observations, the samples were covered with a thin layer of gold in a Leica EM ACE 200 low-vacuum coater (Wetzlar, Germany). A high-resolution Phenom ProX SEM microscope (PhenomWorld, Eindhoven, The Netherlands), operated in a backscattered electron mode, was used for the observations.

**Figure 2:**
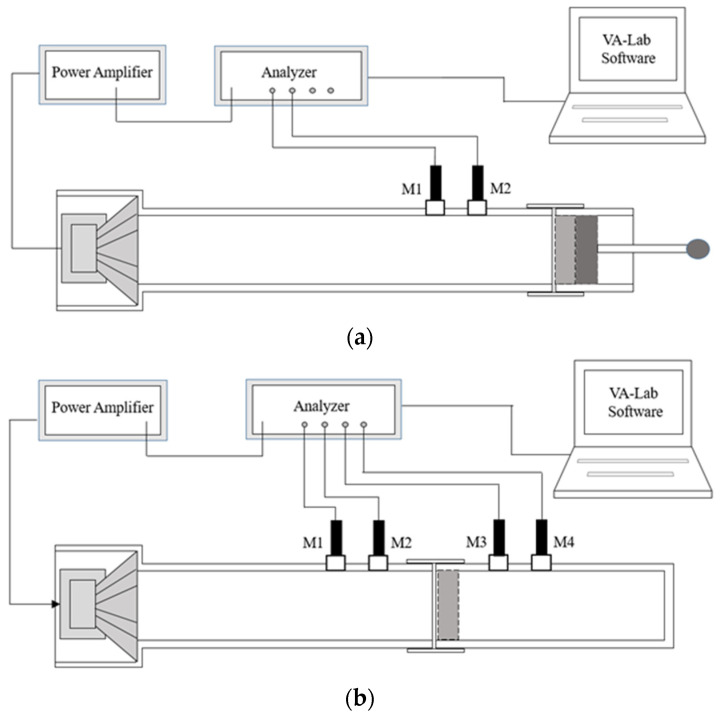
Scheme of experimental setup used for acoustic measurements of (**a**) sound absorption coefficient measurement, (**b**) sound transmission loss measurement.

The acoustic properties of the pile carpets and nonwovens used as carpet underlayment were characterised by the normal incidence sound absorption coefficient (SAC) and normal incidence sound transmission loss (STL). The parameters were measured according to the relevant standards, PN-EN ISO 10534-2 and ASTM E2611, using the BSWA impedance tube system SW422+SW477 (BSWA Technology Co., Ltd., Beijing, China). The measuring stand included two impedance tubes with inner diameters of 30 mm (SW422) and 100 mm (SW477), MI 19 microphones (¼ ’’ ICP), a MC3242 analyser, and a PA50 amplifier. A scheme of system configurations for the measurement of the sound absorption coefficient (SAC) and sound transmission loss (STL) is presented in Figure 2. 

The measurements were carried out at the incident wave’s one-third-octave band frequencies within the 80–5000 Hz range. Two measurements were performed for each sample, and mean values were calculated. Based on the measurement results, the sound absorption average (SAA) was calculated as the arithmetic mean of the sound absorption coefficients for the twelve one-third-octave bands from 200 to 2500 Hz. 

## 3. Results

### 3.1. Acoustic Properties of Nonwovens Used as Carpet Underlayment

The nonwovens used as carpet underlayment are characterised by the parameters presented in Table 1. The mass per unit area of the wool nonwovens was from 200 to 400 g/m^2^. The thickness of the nonwoven with the lowest mass equalled only 3.6 mm. For the nonwovens with higher mass, the thickness was 1.5 mm higher. The characteristics of the woollen nonwoven with the lowest mass corresponds well with the parameters of the polyester nonwoven, which is ordinarily used as underlayment in commercial products. 

**Figure 3:**
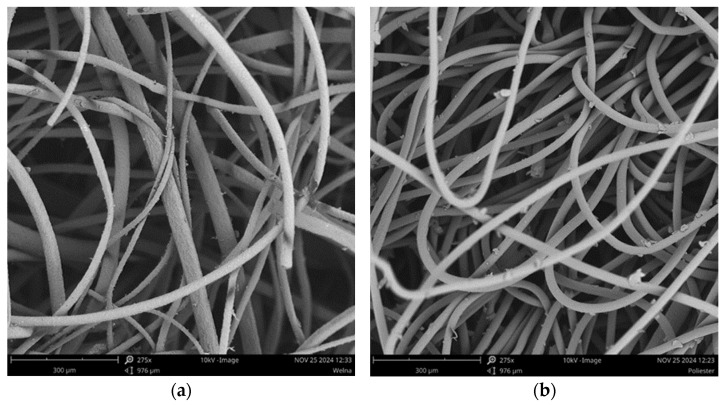
SEM images of nonwovens made from (**a**) wool and (**b**) polyester.

Figure 3 presents the SEM images of the nonwovens. As a waste of the tanning industry, the wool is a mixture of thinner and coarser fibres with diameters between 18 and 50 µm. The thinner fibres are flexible and easily bent, forming an entangled structure. The coarser and stiffer fibres are incorporated into a tangled network as straight fibres without bends. The surface of the wool fibres is covered with characteristic scales. The scales facilitate the mutual interlocking of the fibres, and the creation of a compact felt structure. 

Unlike wool, polyester fibres are uniform, with a diameter of 25 µm. The fibres are sufficiently flexible to form a tangled web. The fibres possess a smooth surface without scales, grooves, or other irregularities affecting the surface roughness. Due to their smooth surface, the fibres can slide easily against each other, so the particular fibres do not stick together and are well separated. 

**Figure 4:**
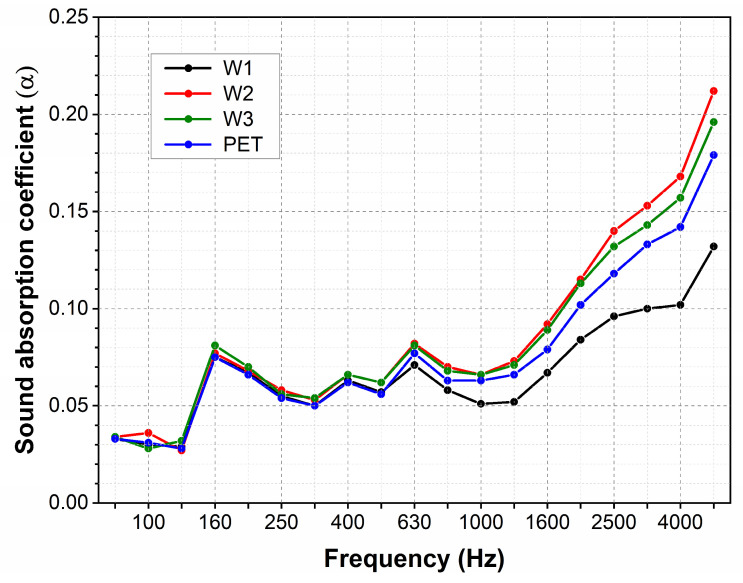
Sound absorption of wool and polyester nonwovens designed for carpet underlayment.

The absorption coefficients measured for the nonwovens change with the sound wave frequency from 0.03 to 0.2 (Figure 4). At low and medium frequencies up to 1600 Hz, the sound absorption coefficient is low and does not exceed 0.1. The SAC slightly fluctuates in this range and is almost the same for all the investigated fabrics. At higher frequencies above 1600 Hz, with increasing frequency, the SAC increases gradually, reaching, for all fabrics, a maximum for the highest frequency of 5000 Hz. For the two wool nonwovens with a higher mass (300 and 400 g/m^2^), the maximal value of the SAC equalled 0.2 is approximately the same as the coefficient measured for the nonwoven polyester. The maximal SAC is ca. 25 % lower for the wool nonwoven with the lowest mass (200 g/m^2^). 

**Figure 5:**
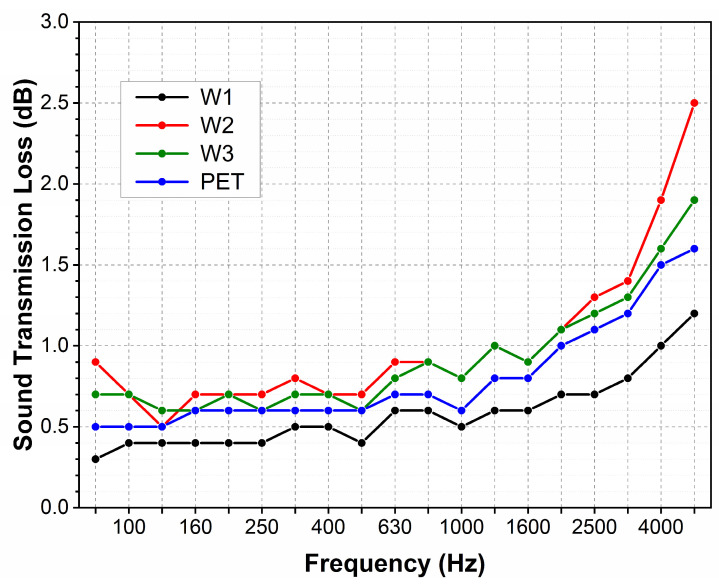
Sound transmission loss of wool and polyester nonwovens designed for carpet underlayment.

The sound transmission loss (STL) for all nonwovens in the whole frequency range takes very low values (Figure 5).

The STL remains below 1 dB at low and medium frequencies. The STL takes on slightly higher values at the higher frequencies, reaching a maximum value at the end of the measurement range. The maximal value for the wool nonwoven with the lowest mass (200 g/m^2^) is slightly lower than that of polyester. For the two wool nonwovens with higher masses (300 and 400 g/m^2^), the STL is almost 1 dB higher.

### 3.2. Sound Absorption of Carpets with Cut and Loop Piles

**Figure 6:**
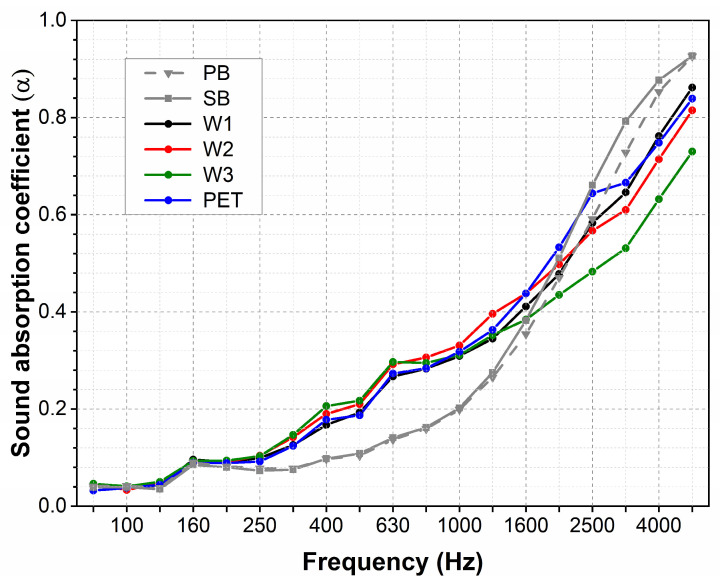
Sound absorption of carpets with cut piles. (PB: primary backing; SB: secondary backing).

For carpets with cut piles, the absorption coefficients accomplished values of 0.04 to 0.9 (Figure 6). In the range of low frequencies until 250 Hz, for all samples with and without the underlayment, the SAC achieves a low value below 0.1. At medium frequencies from 250 to 1600 Hz, the SAC takes on increasingly larger values, reaching a value of 0.4 at the end of this range. In the first range of medium frequencies from 250 to 630 Hz, the increments in the SAC are minimal for the carpets containing only the primary (PB) and secondary backing (SB). For samples coupled with underlayment, independently of the underlayment type, the increments in the SAC in this range are about 0.1 higher. A further increase in the SAC is observed for higher frequencies above 1600 Hz for all samples. For samples coupled with the underlayment, the SAC achieves a maximum of 0.85. The SAC value (0.9) is slightly higher for samples containing only primary and secondary backings.

**Figure 7:**
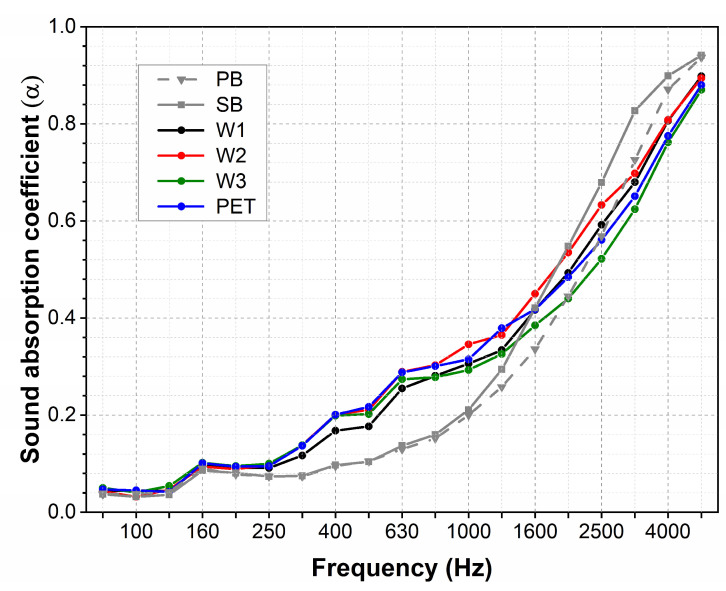
Sound absorption of carpets with loop piles.

For loop pile carpets, the values of the absorption coefficients are very similar to those measured for carpets with cut piles (Figure 7). At low frequencies, the SAC achieves a value below 0.1, and then, with increasing frequency, it reaches increasingly higher values up to 0.9 for the highest frequency of 5000 Hz. Similarly, as for cut pile samples, the SAC of samples coupled with the underlayment is higher at medium frequencies. The addition of the underlayment caused the SAC values to be approximately 0.1 higher in this range.

**Figure 8:**
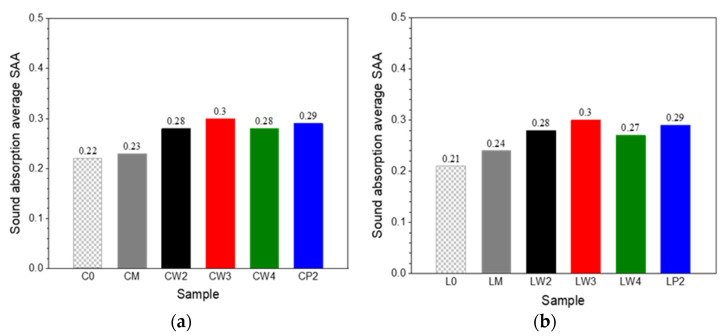
The sound absorption average SAA of tufted carpets with (**a**) cut pile and (**b**) loop pile.

For carpets with cut piles containing only the primary backing, the sound absorption average SAA equals 0.22 (Figure 8). For samples containing the secondary backing layer, the SAA is minimally higher. The SAA is approximately 25 % higher for carpets coupled with the underlayment. The SAA varies between 0.28 and 0.3 for carpets with the underlayment made from polyester and wool, independently of the nonwoven material and weight. For carpets with loop piles formed with and without underlayment, the SAA values are practically the same as those with cut piles. 

### 3.3. Sound Transmission Loss of Carpets with Cut and Loop Piles

**Figure 9:**
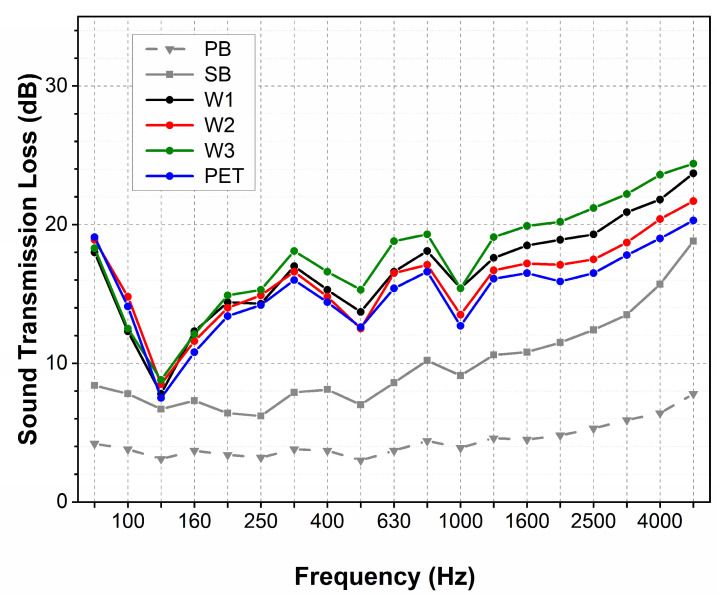
Sound transmission loss of carpets with cut piles.

The sound transmission loss (STL) for carpets with cut piles varies between 5 and 25 dB (Figure 9). The STL is low for the sample with only the primary backing, reaching merely 5 dB for almost all frequencies. The STL is significantly higher for the sample with the base built from primary and secondary backings. In this case, for the low and medium frequencies, the STL fluctuates around 8 dB and then, at higher and higher frequencies, it becomes larger and larger, reaching 20 dB at the highest frequency of 5000 Hz. The STL of carpets coupled with the underlayment for low and medium frequencies varies between 12 and 18 dB. It becomes higher at higher frequencies and achieves a maximal value of 25 dB at the highest frequency.

For all frequencies, the STL of carpets coupled with the underlayment is approximately 10 dB higher than the samples containing only the carpet base. The STL values measured for carpets with different underlayments at low and medium frequencies are identical, independent of the underlayment material and unit weight. The STL is ca. 5 dB higher for wool underlayment samples at higher frequencies. 

**Figure 10:**
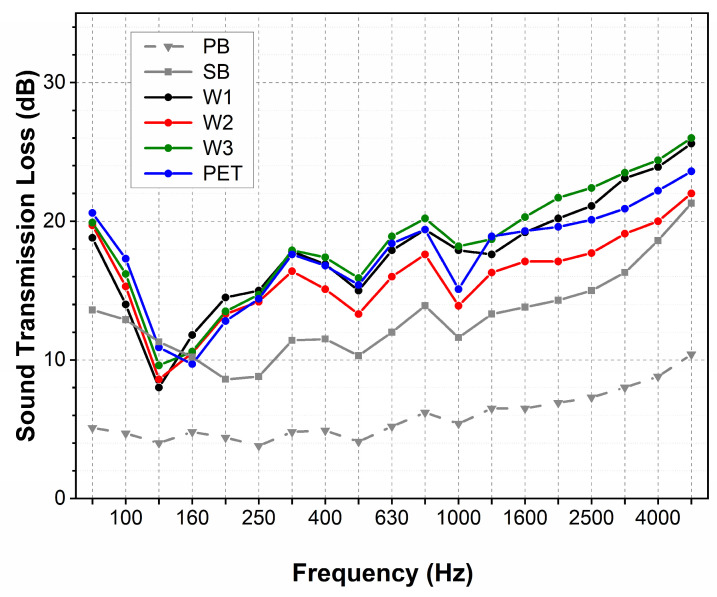
Sound transition loss of carpets with loop piles.

Similarly to cut-pile carpets, the STL measured for carpets with loop piles varies between 5 and 25 dB (Figure 10). Analogously, the STL is very low for the sample containing only the primary backing. The STL reaches values between 10 and 20 dB for the sample with the base constructed from primary and secondary backings. This value is higher than the STL for carpets with cut piles. 

For carpets coupled with the additional underlayment, the STL varies between 12 and 18 dB at the low and medium frequencies. For these samples, the STL has the highest value of 25 dB at the highest frequency. These values are approximately 15 dB higher than values determined for the carpet with primary backing, and 10 dB higher compared to the carpet with two backings. 

Regardless of the type of material, the STL of the carpets coupled with different underlayments has the same value for low and medium frequencies. Similarly to the cut piles, at higher frequencies, for samples containing the wool underlayment, the STL is ca. 5 dB higher than for the sample with two backings, and 10 dB higher than for the sample with primary backing.

## 4. Discussion

For carpet underlayment, the nonwovens made from wool and polyester were applied. The nonwovens were manufactured using a needle-punching technique commonly used to produce nonwoven materials. During manufacturing, the punched needles pass through a voluminous fibrous web, forming a compact structure of mechanically entangled and interlocked fibres. For both types of nonwovens, their structure consists of many built-in tortuous capillary channels and interconnected pores, which provide the nonwoven with high accessibility to the sound wave. The incidental sound waves entering and passing the nonwoven cause the air molecules accumulated within pores to vibrate. As a result of friction, the oscillating air molecules lose their energy, transforming the sound wave energy into thermal and viscous heat.

Like typical porous absorbers, the nonwovens reveal their lowest absorption capacity at low and medium sound wave frequencies [31]. Part of this range of frequencies, from 60 to 250 Hz, falls on the normal speaking vocal range [32]. For high frequencies, the energy loss of sound waves in the nonwovens occurs more intensively, so the absorption coefficients achieve higher values.

The determined coefficients for the investigated nonwovens are relatively low, and lower than those obtained for needle-punched nonwovens manufactured from wool or polyester fibres examined in other studies [33,34]. It is known that the sound absorption capacity of the nonwovens depends on several parameters, including manufacturing method, fibre characteristics, thickness, mass per unit area, porosity, tortuosity, and other structural parameters [35,36,37,38]. The low absorption of the investigated nonwovens resulted from their less-dense structure and relatively low thickness. 

The wool nonwovens’ absorption coefficients across the whole frequency range are similar to those determined for the nonwoven made from polyester fibres. Despite the essential differentiation in surface characteristics between wool and polyester fibres, the sound absorption capacity for both nonwovens is indistinguishable, regardless of the fibre type. Their efficiency in sound absorption is also almost identical despite the minor discrepancies in the nonwovens’ thickness and mass per unit area. 

Across the whole frequency range, the sound transmission loss of nonwovens achieved very low values. The sporadic papers on the transmission loss of nonwovens report that sound transmission loss is most influenced by the weight of the initial carded web and increases with its increasing weight [39]. The investigated nonwovens actually possess low weight, and, due to low needle-punching density and low thickness, their ability to insulate sound is low.

Adding the underlayment to the pile carpets’ base layer forms a three-layered structure, significantly changing the carpets’ sound absorption capacity and considerably influencing its sound transmission. The underlayment possesses a structure and geometry different from the piles’ and base layers’ and reacts differently to incident sound waves. A random web of fibres in the nonwovens forming the underlayment creates more tortuous channels, increasing the sound absorption and reducing the sound-wave energy transmitted through the material more effectively. Consequently, carpets’ acoustic performance strongly depends on the characteristics of separate layers.

Coupling the underlayment obviously increases the carpet thickness, increasing the path length along which the sound wave travels through the carpet. Due to the extension of the path, more sound energy is converted into thermal and viscous heat. The enhancement of sound absorption by increasing material thickness by adding nonwoven layers is a common practice and was repeatedly observed for multilayered structures [40,41,42]. 

Increasing the carpet’s thickness by coupling an underlayment does not impact the carpet’s absorption capacity at low sound-wave frequencies. In the low frequencies range, the sound absorption of carpets coupled with the underlayment is the same as that of carpets without underlayment. The positive impact of the underlayment on sound absorption is observed at medium frequencies. The absorption coefficients determined for carpets coupled with the underlayment are significantly greater in this range. This is observed for underlayments made from wool and polyester. For the higher frequencies, the impact of the underlayment on the absorption capacity becomes again imperceptible. For all samples with and without underlayment, the absorption coefficients were high and only slightly differed from each other.

While the underlayment’s influence on carpet sound absorption is observed only at medium frequencies, its impact on transmission loss was observed across the whole frequency range. A significant increase in transmission loss across the whole frequency range was observed for carpets with a base constructed with two backing layers. Adding the thin second backing for carpets with cut and loop piles increases transmission loss by 5 and 8 dB, respectively. The coupling of the additional underlayment leads to a further increment of the transmission loss, for carpets with both cut and loop piles. 

The observed increase in the STL for a carpet made of three layers is close to 10 dB. Such an increase caused by the addition of the underlayment is much greater than the STL of the nonwovens used as carpet underlayment alone. The cumulative STL of the carpet is greater than the sum of the STLs for the individual layers. 

The transmission loss of carpets, coupled with the underlayment, reached 20 dB. Compared to other studies, this level is two times smaller than the transmission loss of the composite acoustic panels, but it is two times greater than that of the assembly of loose wool or cotton fibres [43]. 

A similar increase in transmission loss is observed for carpets coupled with each underlayment, regardless of the fibre type and nonwoven parameters.

## 5. Conclusions

Applying a nonwoven as carpet underlayment provides better sound control in the room. The underlayment improves the carpet’s sound absorption only at medium sound wave frequencies. At low and high frequencies, the impact of the underlayment on the carpet’s sound absorption is invisible. The coupling of the underlayment significantly improves the sound insulation. The transmission loss is almost two times greater for carpets coupled with the underlayment across the whole frequency range. Carpets with an underlayment made from a wool nonwoven may efficiently attenuate noise and reduce its transmission across the floor.

The acoustic performance of the nonwoven made from waste wool obtained from the tannery industry was the same as that of the polyester nonwoven. Adding wool underlayment improves the acoustic characteristics of the carpets to the same extent as the underlayment made from polyester. 

Considering acoustic performance, wool nonwovens can be an effective, eco-friendly, sound-absorbing carpet underlayment, a reasonable alternative to commonly used products from synthetic fibres. Under the zero-waste strategy, using wool for manufacturing underlayments may reduce the amount of wool waste and improve its utilisation. 

Using wool underlayment enables the manufacturing of carpets in which the two layers, the pile layer and underlayment, are made from sustainable natural fibres. In this way, synthetic fibres are limited to a thin layer of the carpet base, which significantly contributes to the decrease in the pollution of the environment with plastic residues. 

The use of nonwoven fabric made from tannery wool waste may involve certain inconveniences resulting from the unique characteristics of the wool. Potential disadvantages, such as low moth resistance, are a challenge that can be overcome with further research.

## Figures and Tables

**Table 1:** Specifications of nonwovens used as carpet underlayment.

Symbol	Material	Mass per Unit Area, g·m^−2^	Thickness, mm
W1	wool	200 ± 25	3.6 ± 0.3
W2	wool	300 ± 30	4.9 ± 0.4
W3	wool	400 ± 40	5.2 ± 0.5
PET	polyester	200 ± 20	4.1 ± 0.3

## Data Availability

The raw data supporting the conclusions of this article will be made available by the authors on request.
